# Surgical outcomes research in LMICs: a narrative review

**DOI:** 10.4314/ahs.v25i1.48

**Published:** 2025-03

**Authors:** Taylor Jaraczewski, Thomas Diehl, Dawda Jawara, Girma Tefera, Syed Nabeel Zafar

**Affiliations:** 1 Department of Surgery, Medical College of Wisconsin, 8701 Watertown Plank Rd., Milwaukee, WI, 53226, USA; 2 Department of Surgery, University of Wisconsin-Madison School of Medicine and Public Health, 600 Highland Ave., Madison, WI, 53792, USA

**Keywords:** Surgical outcomes, research, LMICs

## Abstract

**Background:**

Surgical outcomes research is sparse in low- and middle-income countries (LMICs). This is due to poor funding, lack of human resources, and inadequate infrastructure. However, a growing number of collaborative small collection of large multinational and multicentered studies have been successfully performed. These studies have overcome regulatory and logistical hurdles and have shown that collecting such data in the LMIC setting is possible.underscore the drive and capabilities of LMIC researchers.

**Methods:**

A review of the literature using PubMed was performed for multicenter and multinational studies on surgical outcomes in LMICs.

**Results:**

All studies collected a diverse array of postoperative outcomes including complications and mortality. Multiple studies performed adjusted analyses to allow for identification of independent risk factors of surgical outcomes. Each study reinforced that outcomes in LMICs are markedly worse than in HICs.

**Conclusion:**

These studies showed that outcomes research is feasible and needed in LMICs. In this review we summarize each of these impactful studies and present strengths, weaknesses, commonalities and gaps that remain.

## Background

Surgical outcomes in low- and middle-income countries (LMICs) are significantly worse than those in high-income countries (HICs). Despite performing only 6.5% of surgeries worldwide, LMICs account for 50% of perioperative mortality.^1^ However, the true extent of this problem is not fully understood. Surgical outcomes data allows health care systems to analyze patient outcomes after specific procedures including in-hospital characteristics, morbidity, and mortality rates. Such data can be utilized to identify disparities. Despite the importance of this data, collection in LMICs is sparse. This lack of data presents a significant barrier to defining and improving surgical outcomes in LMICs.

Data collection in LMICs is challenging due to a reliance on paper-based medical records, lack of standardized record keeping, poor infrastructure, low quality and incomplete documentation, and limited human resources.^2^,^3^ Currently, metrics used to measure effective care in LMICs include procedural volume, workforce availability, knowledge transfer to trainees and facility accessibility.^4^ While important for assessing access, these measures do not give adequate insight into surgical outcomes.^5^ Important outcomes metrics include clinically based findings such as complication rates, 30-day readmission, and mortality. Despite barriers to data acquisition, several large prospective multinational multicentered studies have successfully captured surgical outcome data in LMICs. In this manuscript we review six of these studies, all of which met our inclusion criteria of being multinational, multicenter, studies on surgical outcomes in LMICs. We aim to summarize methods, results, strengths, and weaknesses, describe commonalities, highlight challenges, successes, barriers and identify remaining gaps.

### Study Description and Selection

We identified six multicenter, multinational studies on surgical outcomes in LMICs. These studies are described below and summarized in Table 1.

**Table 1:** Overview of each of the studies included in the narrative review

Study	Year	Methods	Study Population	Study Participants	Outcome Measures	Country Income Level Breakdown
European Surgical Outcomes Study (EuSOS)	2011	Prospective, cohort study 7 days data collection	Adult (>16yrs), non-cardiac surgical patients	498 hospitals 28 European nations N = 46,539	Primary: In-hospital mortality Secondary: LOS, ICU admission, duration critical care stay	7.4% (N=2) Upper Middle 92.6% (N=25) High
International Surgical Outcomes Study (ISOS)	2015	Prospective, cohort study 7 days data collection	Adults (≥18yrs) elective surgical patients with planned overnight stay	498 hospitals 27 countries (19 high-, 7 middle- and 1 low-income) N = 44, 814	Primary: In-hospital complications Secondary: In-hospital mortality, failure to rescue	3.7% (N=1) Low 25.9% (N=7) Middle 70.3% (N=19) High
GlobalSurg1	2016	Prospective, cohort study 14 days data collection	Any patient undergoing emergency intraperitoneal surgery (no age restrictions)	357 hospitals 58 countries N = 10,745 High: 6,538 Middle: 2,889 Low: 1,318	Primary: 24h postoperative mortality rate Secondary: 30-day mortality rate, postop complications and reintervention rate	12.3% (N=1,318) Low* 26.9% (N=2,889) Middle* 60.8% (N=6,538) High*
African Surgical Outcomes Study (ASOS)	2018	Prospective, cohort study 7 days data collection	Adults (>18yrs) undergoing inpatient surgery (elective and urgent/emergent)	247 hospitals 25 African countries Middle: 11 Low: 14 N = 11,422	Primary: In-hospital postop complications Secondary: In-hospital mortality	56% (N=14) Low 44% (N=11) Middle
GlobalSurg2	2018	Prospective, cohort study 14 days data collection	Any patient undergoing elective or emergency surgery including gastrointestinal resection (no age restrictions)	343 hospitals 66 countries N= 12,539 High: 7,339 (193 hospitals in 30 countries) Middle: 3,918 (82 hospitals in 18 countries) Low: 1,282 (68 hospitals in 18 countries).	Primary: 30-day SSI rate Secondary: 30-day postop mortality rate, periop antibiotic administration, 30-day postop reintervention rate, prevalence of antimicrobial resistance for SSI, in-hospital SSI incidence, 30-day SSI incidence	27.3% (N=18) Low 27.3% (N=18) Middle 45.4% (N=30) High
GlobalSurg3	2021	Prospective, cohort study 28 days data collection	Adults (>18yrs) undergoing surgery for primary breast, colorectal, or gastric cancer	428 hospitals 82 countries N = 15,958 High: 9,106 Upper-middle: 2,721 Low- or lower-middle: 4,131	Primary: 30-day postoperative mortality and 30-day major complication Secondary: 30-day complication, 30-day unplanned reintervention or readmission, cancer-specific complications, cancer treatment pathways	34.1% (N=28) Lower-middle 28% (N=23) Upper-middle 37.8% (N=31) High

LOS = length of stay, ICU = intensive care unit, SSI = surgical site infection.

* Data represented as number of patients from respective country income levels.

### European Surgical Outcomes Study (EuSOS)

Conducted in April 2011 the EuSOS is a multinational cohort study of European adult (>16 years), non-cardiac surgical patients undergoing elective or non-elective inpatient surgery and was one of the first cross-sectional cohort studies for surgical outcomes.^6^ The primary outcome was in-hospital mortality. Secondary outcomes included: duration of hospital stay, planned admission to critical care, unplanned admission to critical care, duration of critical care stay and 28-day mortality. The study collected data from 28 European countries and reported overall 4% in-hospital mortality, ranging from 1.2% [95% CI 0·0–3·0] in Iceland to 21.5% [16·9–26·2] in Latvia. Variables independently associated with mortality included country where surgery was performed, grade of surgery, surgical procedure category, urgency, American Society of Anesthesiologists (ASA) score, age, metastatic disease, and cirrhosis. The EuSOS findings are valuable as they describe the mortality disparity between different countries. However, the study did not assess post-operative complications or stratify by income. Hospitals were sampled via a voluntary convenience sample; thus, the study population was likely biased and not representative of the entire population. Further, some countries included in the study have raised questions in regards to the methodology because internal mortality rates were significantly different than those presented in the study.^7^

### International Surgical Outcomes Study (ISOS)

In 2015, the ISOS sought to assess the global incidence of complications, associated risk factors, and mortality following elective inpatient surgery.^8^ Their study population included a 7-day international cohort of 44,814 adult (age ≥ 18) surgical patients from 474 hospitals in 19 high-, 7 middle- and 1 low-income country. The ISOS found that overall 16.8% of patients developed in-hospital complications, and 0.5% patients died before hospital discharge. Overall failure to rescue – defined as mortality among patients who experience complications - was 2.8%. The most common complications were postoperative bleed (N=1,362, 3%), superficial surgical site infection (SSI, N=1,320, 2.9%), and arrhythmia (N=1,222, 2.7%). Interestingly, when stratified by country income level, LMICs reported younger patients with lower ASA scores, lower complication rates than HICs (11.1% versus 19.8%), and slightly lower mortality overall (0.4% versus 0.5%), but higher failure-to-rescue (3.3% vs. 2.6%). LMICs also had less planned postoperative admission to critical care units compared to HICs (6.9% vs 11.4%) as well as less critical care admissions to treat complications (2% vs 3.2%). These findings broadly suggest differences in LMIC postoperative care, which affect the ability to rapidly identify and treat complications when they occur.^8^,^9^ Subsequent ISOS offshoot studies delved further into the concept of failure-to-rescue and discovered that when stratifying hospitals by quintiles of surgical case volume, there was a 3-fold difference in mortality (Q1: 0.6% vs Q5: 0.2%) and two-fold difference in capacity to rescue (Q1: 3.6% vs Q5: 1.7%).^10^ The authors conclude that dissimilar failure-to-rescue rates across healthcare systems suggest the presence of preventable deaths and opportunity for intervention.^26^ While the results may be biased by differences in post-operative monitoring between HICs and LMICs, specifically poor postoperative complication measurements in LMICs, the ISOS contributed important data concerning failure-to-rescue following surgical complications.

### Global Surgical Outcomes Collaboration

In recent years, the National Institute for Health Research's (NIHR) Global Health Research Unit on Global Surgery has become a leader in large collaborative projects investigating global surgical outcomes. Seeking to better understand research priorities from leadership in LMICs, the collaboration conducted an LMIC-led modified Delphi process involving global research hubs, leading to identification of three priority research topics: (1) access to surgery, (2) outcomes of cancer surgery, and (3) perioperative care.^11^ In the following years, they created the Global Surgical Outcomes Collaboration (GlobalSurg Collaborative), which has published three cross-sectional, cohort studies on global surgical outcomes.

### GlobalSurgl: “Mortality of emergency abdominal surgery in high-, middle- and low-income countries”

The GlobalSurg1 study sought to assess differences in 24-hour and 30-day mortality rates after emergency abdominal surgeries.^12^ Collecting operative data from 357 centers across 58 countries (10,745 patient records, 60.8% from high-HDI vs 12.3% from low-HDI), the study found post-operative 24-hour mortality to be three times higher in low- compared with high-Human Development Index (HDI) countries, even after adjusting for prognostic factors (24-hour mortality: 1.1% in high-HDI countries, 1.9% in middle-HDI countries, 3.4% in low-HDI countries). Similarly, 30-day post-operative mortality in low-HDI countries was nearly twice that of high-HDI countries (4.5% in high-HDI countries, 6.0% in middle-HDI countries, 8.6% in low-HDI countries).

The GlobalSurg1 study uncovered stark disparities in mortality outcomes following emergency abdominal surgeries. In addition, through methods employing a “collaborative bedside network,” the study demonstrated that it is feasible to collect post-operative mortality statistics, even in low-HDI countries.

### GlobalSurg2: “Surgical site infection after gastrointestinal surgery in high-income, middle-income, and low-income countries: a prospective, international, multicentre cohort study”

In the follow-up GlobalSurg2 study, investigators sought to determine global variability in surgical site infection (SSI) rates.^13^ They collected patient-level data for 12,539 patients from 343 hospitals in 66 countries. GlobalSurg2 concluded that the incidence of SSI varied significantly across income levels (9.4% in high-HDI countries, 14% in middle-HDI countries, and 23.2% in low-HDI countries). One weakness of this study was a study population heavily skewed toward HIC countries (58.5% high-HDI versus 10.2% low-HDI).

### GlobalSurg3

The GlobalSurg3 study's primary outcomes were post-operative mortality and major complication rate 30 days after cancer surgery.^14^ They included 15,958 charts of which 9,106 (57.1%) were from high-income countries, 2,721 (17.0%) upper-middle income, and 4,131 (25.9%) were from lower-middle income. The authors analyzed outcomes after gastric, colorectal, and breast cancer surgery from 428 hospitals in 82 countries and found that 30-day postoperative mortality is up to four times higher in LMICs, compared to HICs, despite similar major complication rates. Specifically, colorectal cancer patients in low-income or lower-middle income countries, were 4.59 (95% CI 2.39-8.80) times more likely to die within 30 days of surgery, compared to patients from high-incomes countries. Similarly, 30-day mortality was substantially higher after gastric cancer resections (OR=3.72, 95% CI=1.70-8.16) in LMICs, compared to HICs. Expanding on the failure-to-rescue findings from the ISOS, GlobalSurg3 found that 30-day mortality rates for patients who experience a major complication was up to five times higher in LMICs than in HICs. This final point was an important conclusion of the GlobalSurg3 study -although the rate and pattern of surgical complications were similar between LMICs and HIC, mortality was several-fold higher in LMICs. Furthermore, later-stage disease at presentation accounted for only a portion of this difference in postoperative mortality.

### African Surgical Outcomes Study (ASOS)

Published in 2018, the ASOS was a multicenter, observational cohort study which sought to determine postoperative in-hospital complications and mortality rates for adults undergoing inpatient surgery (both elective and non-elective).^15^ In total, 11,422 patients were included from 247 hospitals across 25 African countries (14 low-income countries, 11 middle-income countries). Postoperative complications and in-hospital deaths were reported in 18.2% and 2.1% of patients respectively. In this study population, infection was the most common postoperative complication, occurring in about 10% of cases. The study concluded that African surgical patients are twice as likely to die after surgery, despite being younger, lower-risk and having fewer complications (compared to the ISOS cohort). In a follow-up cluster-randomized controlled trial, ASOS2, investigators tested whether a package of enhanced postoperative surveillance interventions could reduce 30-day in-hospital mortality. The ASOS2 concluded that implementation of an intervention package did not decrease 30-day in-hospital mortality among surgical patients and additional research is needed to identify potential solutions for preventing death from surgical complications in Africa.^16^

## Discussion

Our study presents a narrative review of the six international multicenter prospective cohort studies on surgical outcomes within LMICs. These studies all had a large sample size, ranging from 10,745 to 46,539 patients, and included a total of 142,897 patients. Each study included a diverse range of countries, from 25 to 82, and included low, middle-, and high-income countries. The number of unique hospitals ranged from 247 to 498. We can glean several insights from these studies.

First, these studies show that it is feasible to collect high-quality, prospective data on surgical outcomes in the LMIC setting. Not only do they prove that it is possible to obtain such data but also it is possible to do at a multi-center and cross-country scale while navigating different regulatory and ethical requirements. However, it should be noted that not all invited institutions participated in each of the studies, which introduces an element of selection bias.

An impressive aspect of all of these studies is the ability to collect a large diverse array of both pre-operative variables and postoperative outcomes. All studies included some combination of pre-operative variables including demographics, comorbid conditions and operative characteristics. These data points are essential for both understanding factors associated with outcomes as well as providing risk adjusted rates that account for differences in severity and case mix. Demographics data that was unanimously collected included age, sex, smoking status, and ASA score. While some operative data was collected in all studies, most only included urgency and/or grade. GlobalSurg1 included the most robust operative data including variables such as start time, bowel resection, surgeon/anesthetist seniority, stoma formation, use of the WHO surgery safety check list, administration of prophylactic antibiotics, and anesthesia type. Performance of a CT scan was only mentioned in GlobalSurg1 and lab values were not discussed in any study. A number of primary and secondary outcomes were measured and are summarized in table 1. All studies collected postoperative complications, though to different degrees. Postoperative mortality rates (POMR) were collected in all studies in the form of in-hospital, 24hour, and/or 30-day time frames. Successful collection of POMR is one of the six core indicators for monitoring access to safe affordable surgical care put forth by the Lancet Commission on Global Surgery (LCoGS), thus it is promising that it was able to be collected by all studies.^4^ It should be noted that GlobalSurg1-3 studies collected the 30-day mortality and complications data. Loss to follow up after hospital discharge is a common barrier in LMICs. Thus, the ability to collect 30-day outcomes has important implications towards accurate assessment of postoperative morbidity and future quality improvement efforts.

Both the ASOS and ISOS studies mention that patients from LMICs were younger and had lower ASA scores compared to HICs and global averages; however, outcomes in LMICs were worse than HICs. This discrepancy between LMICs and HICs was apparent throughout all of the studies. One example of this is failure-to-rescue in LMICs, which recurred in 3 of the studies. The ISOS study presented lower complication rates in LMICs compared to HICs, but with higher failure-to-rescue. These findings were mirrored in cancer patients in the GlobalSurg3 study, which showed five-times higher mortality rates in patients with complications in LMICs vs HICs in the setting of comparable mortality rates. Failure-to-rescue is a litmus test for the resilience of a healthcare system and has been utilized as a quality metric in other systems.^17^ Causes of poor failure-to-rescue are multifactorial and includes patient-level, operative, and hospital factors. Importantly, ASOS2 showed that it was not solely due to delayed diagnosis of complications. Further research targeted towards failure-to-rescue has the potential to significantly decrease the burden of postoperative morbidity in LMICs.

Mortality outcomes were also worse in lower compared to higher income countries. GlobalSurg1 showed that mortality was three times higher in low-compared to high-HDI countries. While the ASOS did not include HICs, the in-hospital mortality rate was 2.1%, which is nearly double that of the HICs in GlobalSurg1. While both studies were quite different it should be noted that GlobalSurg1 included only patients undergoing emergency cases whereas ASOS had only 33.4% emergency included both elective and urgent/emergent. Further, of the patients in ASOS who died, only 47.9% had undergone emergency surgery. More stunning is the fact that 20.1% of patients who died had undergone an elective case. This is just one example that highlights the severe disparities in surgical outcomes between HICs and LMICs. Additionally, it further reinforces the idea that surgical outcomes research needs to expand in LMICs to facilitate the next step, which are quality improvement programs.

A number of studies performed analyses to determine factors associated with outcomes. Both ASOS and GlobalSurg1 utilized multivariable logistic regression to assess factors independently associated with mortality. ASA score was found to be associated with postoperative in-hospital (ASOS), 24-hour, and 30-day mortality (GlobalSurg1). In addition to ASA score, ASOS showed that surgical urgency and grade were associated with in-hospital mortality. GlobalSurg1 found that age and diagnosis were associated with 24-hour mortality and country HDI and diagnosis were associated with 30-day mortality. Alternatively, use of a surgical safety checklist was associated with reduced mortality at 30 days in GlobalSurg1. Similar to GlobalSurg1, GlobalSurg2 found that lower HDI was associated with higher SSI rates regardless of the contamination level. Further, presence of SSI was associated with increased 30-day mortality, 30-day intervention, organ space infection, other health care associated infections, and median length of stay. GlobalSurg3 also found an association between country income level and patient outcomes. Specifically, 30-day mortality was higher in gastric cancer in low- or lower-middle income countries as well as in colorectal cancer in upper-middle income countries. Additionally, the proportion of patients dying following a major complication was higher in LMICs compared to HICs. However, the presence of postoperative care infrastructure provided a protective effect in LMICs. A nested analysis showed that patient performance status and emergency surgery were associated with death following major complication. All of these findings suggest that country income status has significant impact on population health outcomes.

Though impactful, many weaknesses can be identified throughout these studies. First despite large patient populations, three studies had fairly low LMIC representation (1 out of 27 countries in ISOS, 12.3% in GlobalSurg1, and 10.2% in GlobalSurg2). Alternatively, GlobalSurg3 had 25.9% of patients from LMICs and ASOS only took place in low- and middle-income countries. A second major weakness is that all studies were snapshots that occurred over 7-day increments. Thus, these are results at single instance in time and not longitudinal data. The collection of outcome data in a continuous or longitudinal manner is a major gap in LMICs. Such systems are essential to quality improvement and form the backbone of surgical quality improvement (QI) programs. Investment into such data collection systems has led to improved mortality rates, decreased morbidity and increased efficiency in other systems worldwide.^18^-^20^ Some small surgical registries have been successfully implemented in LMICs; however, these have been targeted towards very specific cases and have limited reported outcomes.^21^-^23^ Large scale registries capturing postoperative outcomes for most procedures are essentially nonexistent in LMICs. Barriers to implementation of these registries include lack of human resources to collect and manage data, no electronic medical systems, inaccurate documentation, poor funding, a deficiency in data storage systems, and lack of stakeholder enthusiasm.^24^,^25^ Working towards adapting such registries to the LMIC setting is critical for improving surgical outcomes in the future.

## Limitations

Limitations of this review include that it is a narrative style review. Thus, the methodology for study inclusion was not as standardized or rigorous as other review formats. While we did define inclusion criteria as “multinational, multi-center, and includes LMICs,” the narrative review methodology includes some subjectivity that can lead to selection bias. To minimize this effect, we ensured we had unanimous support of all authors prior to inclusion.

## Conclusion

In this review, we aimed to recognize the significant contributions of LMIC researchers in perioperative outcomes, emphasizing that high-quality data from these settings is not an exception but a testament to the capabilities and leadership of local researchers. We acknowledge the historical imbalance where research from HICs has often dominated the global narrative, and the need to shift towards more equitable, LMIC-led initiatives. While some of these studies were led by outside western organizations, their success hinged upon the local researchers themselves. Despite limitations in financial and human resources, infrastructure, regulatory bodies and challenges with ethical review boards, this review shows that strong leadership and determination from researcher on the ground leads to collection of high-quality surgical outcome data in LMICs. Studies reviewed here have successfully collected postoperative mortality and complications along with enough variables to adequately risk adjust these outcomes for benchmarking. While large-scale studies have provided important insights, we would emphasize the need for these findings to be translated to actionable, context-specific interventions that improve patient care on the ground. It is critical to strengthen local robust outcomes research and ride the inertia of these researchers. Our concluding final themes identified include (i) higher rates of complications and mortality by country income level - LMICs having worse outcomes – despite patients being younger and healthier, (ii) identification of failure-to-rescue as a major targetable area that can substantially improve outcomes and (iii) the need to develop local systems capable of collecting continuous longitudinal surgical outcome data in order to develop surgical QI programs. This is the next step in global surgery and will be imperative for making the jump from identifying areas for improvement to creating systems capable of improving care for patients.
